# Stigma and HIV service access among transfeminine and gender diverse women in South Africa – a narrative analysis of longitudinal qualitative data from the HPTN 071 (PopART) trial

**DOI:** 10.1186/s12889-020-09942-5

**Published:** 2020-12-10

**Authors:** Laing de Villiers, Angelique Thomas, Dionne Jivan, Graeme Hoddinott, James R. Hargreaves, Virginia Bond, Anne Stangl, Peter Bock, Lindsey Reynolds, on behalf of the HPTN 071 (PopART) study team

**Affiliations:** 1 Desmond Tutu TB Centre, Department of Paediatrics and Child Health, Faculty of Medicine and Health Sciences, Stellenbosch University, Stellenbosch University, K-Floor, Clinical Building, Tygerberg Campus, Francie van Zyl Drive, Tygerberg, Cape Town, 7505 South Africa; 2 Department of Social and Environmental Health Research, Centre for Evaluation, London School of Hygiene and Tropical Medicine, London, UK; 3 Zambart, School of Public Health, University of Zambia, Lusaka, Zambia; 4 Department of Global Health and Development, Faculty of Public Health and Policy, London School of Hygiene and Tropical Medicine, London, UK; 5 International Center for Research on Women, Washington, USA; 6 Department of Sociology and Social Anthropology, Faculty of Arts and Social Sciences, Stellenbosch University, Stellenbosch, South Africa

**Keywords:** Transfeminine, Gender diverse, Stigma, HIV service access, South Africa

## Abstract

**Background:**

Transgender women have a disproportionately high HIV prevalence compared to cisgender women and men who have sex with men, which puts them at risk of HIV-related stigma (Baral SD et al., Lancet Infect Dis, 13;3, 2013). People whose gender identities are in tension with dominant social norms (including transgender women) often also experience gender identity-related stigma. There has been increasing attention to transgender people in HIV research and interventions. However, very little research has been done in sub-Saharan African countries.

**Methods:**

We conducted a qualitative cohort study which included eight transfeminine and/or gender diverse women (four living with HIV) in Western Cape, South Africa, for a follow-up period of 12–18 months. Using a narrative analysis approach, we set out to understand how transfeminine and gender diverse participants in the cohort anticipated, experienced and internalised HIV stigma and gender identity stigma, and how these stigmas affected HIV service access.

**Result:**

We found that participants reported anticipated, experienced, and internalised stigma relating both to their gender identity and to living with HIV. Participants reported inconsistent uptake of antiretroviral therapy (ART) services (including ART initiation and adherence) that they linked to stigma. We also found that gender diverse women and transfeminine women are challenged with other stigmatising social identities, like being a sex worker, drug user and/or a man (or assigned male sex at birth) who have sex with men (MSM). We use the terms ‘transfeminine’ and ‘gender diverse’ as terms that are inclusive of gender variant people who were all assigned male sex at birth and identify as women in some or all aspects of their lives. The persons in our study also showed gender identifications that were fluid and sometimes varied in different contexts and situations, therefore gender identity and sexual identity were often conflated for these individuals. Participants managed high levels of reported stigma by drawing on social support networks like families, friends and peers.

**Conclusion:**

Our study provides exploratory work on how stigma may affect HIV services uptake amongst gender diverse women and transfeminine women in South Africa. We recommend future studies to further explore the unique HIV risks of gender diverse individuals.

**Trial registration:**

DOH-27-0513-4253.

**Supplementary Information:**

The online version contains supplementary material available at 10.1186/s12889-020-09942-5.

## Background

There has been increased focus on transgender women in HIV research worldwide [1–3]. A 2013 systematic review on HIV infection burden amongst transgender women concluded that transgender women are 49 times more likely to be living with HIV than the general population [4]. Related to a high risk of HIV, transgender women also face high levels of stigma [5]. Stigma among transgender women is associated with lower access to social and health care services, including HIV prevention, testing and treatment services [4, 6]. The high levels of stigma experienced by transgender women has also been linked to physical and sexual violence, substance use and abuse, low levels of education, sex work, incarceration, poor mental health and limited economic support or resources [1–3].

More recent studies on HIV and stigma amongst transgender people have included intersections of stigma related to HIV, gender identity and sexual orientation (or sexual identity) [7–9], as well as other social group identities like being a sex worker [10, 11]. Researchers have suggested that transgender people face stigma that is intricately connected to various marginalised social identities related to their gender, sex, employment, social class, race, illness, and drug use, and that stigma mitigation interventions must consider such intersectional stigma [12]. In the local South African context these social identities are interconnected with historical political, social and racial discrimination.

Despite growing international concern about the experiences of transgender people, there has been very little research focused on the unique needs of transgender people in South Africa [13]. The lack of transgender-related research is concerning, as South Africa has the largest HIV epidemic in the world and is home to 19% of the global number of people living with HIV [14]. South Africa’s National Strategic Plan for HIV, TB and STI’s 2017–2022 aims to reduce new infections, as well as improve treatment, care and support amongst key and vulnerable populations, explicitly including transgender people [15]. Further, sexual and gender minority groups are visible and legally protected in South Africa, unlike other sub-Saharan African countries. Nonetheless, they face discrimination and stigma and high levels of crime and violence [13].

The existing body of research that has been conducted with transgender people in South Africa has explored topics linked to experiences, barriers and opportunities to accessing public services. Two studies by Müller [16, 17] explored gender minority people’s general struggle against discrimination by health workers, as well as poor provision of health services. Other studies reported on health workers’ experiences in providing services for transgender persons [17–20]. In these studies, poor service provision was linked to health workers’ lack of knowledge about transgender people and health workers’ inexperience of working with transgender people. Some studies have also noted how identity erasure,^1^ for transgender people occurs in other sectors, including education, public services and in the workplace [17, 20–22]. Few studies, however, have reported on the lived experiences of transgender women in South Africa [13]. In research and practice, transgender people are often conflated with lesbian, gay and bisexual groups, emphasising their sexual orientation and neglecting their gender identity [20].

In this study, we want to draw attention to the intersections and nuances of gender identity and sexual identity stigma amongst people who are included on non-binary gender terms, or as transgender. Stryker (2017) uses transgender as a very diverse term to include all/people who are non-fitting to binary gender norms. In accordance to more recent research, we use the term ‘transfeminine’ in combination with ‘gender diverse’, to explore experiences of women who were all assigned male sex at birth, but who have a feminine gender identification in different ways [23].

We explore in this article how stigma affects HIV service access among transfeminine and gender diverse women in four communities in the Western Cape, South Africa. We present a case-comparative analysis of participants’ narratives about stigma and HIV service access in order to understand, firstly, how transfeminine and gender diverse women experience, anticipate and internalise HIV- and gender identity-related stigma. Secondly, we aim to elucidate how stigma affects HIV service access for transfeminine and gender diverse women. Finally, we explore how transfeminine and gender diverse women potentially overcome stigma to access HIV services.

## Methods

### Study design/overview

The data used for this paper comes from a subsample of a qualitative cohort (that included about 90 family or household units), which was administered as a social science component of the HPTN 071 (PopART) trial, a cluster-randomised trial that tested the effects of an HIV prevention package of universal HIV test and treat services on community HIV incidence [24]. The analysis for this subsample does not speak to either main trial outcomes or main social science outcomes. This analysis, however, was framed by a stigma ancillary study to the main trial which aimed to understand the association between HIV-related stigma and uptake of the PopART intervention package [25].

The stigma ancillary study hypothesised that HIV-related stigma may increase, decrease or change amidst a universal test and treat landscape. With this hypothesis in mind, we included key populations at high risk of HIV infection, like MSM, transgender women, sex workers, incarcerated people and migrants in a qualitative cohort. There is growing evidence that the accessibility of HIV treatment might put more strain on people living with HIV, as they are expected to be responsible for their treatment [25]. This might put even heavier strain on people living with HIV who have additional discriminated social identities like being a transgender person. We sought to understand the unique needs of eight transfeminine women and gender diverse women and the tensions experienced around their identities when seeking HIV prevention and care services.

### Conceptual frame

Stigma, as it relates to HIV or gender identity, on an individual-level is generally understood along three manifestations: anticipated (fear of experiencing stigma if one’s gender-identity or HIV status becomes known), experienced (experiences of being treated differently or being discriminated against), and internalised (where the negative views expressed in society are accepted to be true in the self) [25, 26]. For the main part of the analysis, we were interested in the way transfeminine and gender diverse women expressed anticipated, experienced and internalised stigma related to their gender identity and HIV status, and how these processes may have affected HIV services, including testing, treatment or adherence support. Although we were primarily interested in the anticipation, experience and internalisation of gender and HIV-related stigma among participants, it was very clear from the start that their lived experiences were very complex that created additional barriers to care. The narrative pieces were investigated with this conceptual framework.

### Recruitment and participation

The research team purposively approached community members, health facility staff, intervention and community-based trial staff, as well as specialised men’s health clinic facility staff to help identify people who were assigned male sex at birth and who identified as women in some way or another (see Results) at recruitment. For inclusion criteria we followed a common definition of ‘transgender’ as an umbrella term to describe people who have a different gender identification than their assigned sex at birth [27], and also included those whose gender identification were fluid (rather than fixed). We identified thirteen potential participants who we formally recruited into the qualitative cohort. Participation also involved the inclusion of family or household members for a 12 to 18 month period. Five participants were either lost to follow up or opted out of participation, which left us with eight participants from four study communities in South Africa.

### Data collection

The eight participants and various individuals from their families and social networks (households) (see Table 1) were retained for the duration of the qualitative cohort (between January 2016 and October 2017). Data collection with participants and their households entailed a series of intensive interviews and observations, as well as more unplanned visits such as attendance of informal musical performances and modelling shows. Interviews were structured around the following topics investigated during the cohort: family structure and kinship; mobility; how they get by; love, sex and romance; HIV services and care; and ambitions, hopes, and fears. Participants and their households were visited approximately every 6 weeks, with an average of thirteen visits per household. Interviews were voice-recorded and additional data in the form of field notes, photos, participatory research activity outputs and researcher reflection documents were collected. In total, discussions with these participants included 140 h of audio recordings.

**Table 1:** Participant summary

Name (Pseudo-nym)	Age	Racial or ethnic group	Language	Self-identity	Health status	Employment	Shelter & Housing type	Shared household income	Household members (incl. Internal and external family, friends or strangers)
Curella	28	Coloured	Afrikaans	“femgay”	HIV negative & no other physical health concern.	Informal work in admin and retail	Shares the lounge with her step-sister in a shared one-bedroom brick house.	R6000 (US$400) per month	Lives with her mother, step-father and step-sister. Her extended family and grandfather **(consented)** live across the street.
Georgina	27	Coloured	Afrikaans	“moffie” or “gay”	HIV negative & no other physical health concern.	Neighbour-hood watch	Has her own room in a shared two bedroom brick house.	R9000 (US$600) per month	Lives with her mother **(consented)**, father and other extended family members.
Stacey	28	Coloured	Afrikaans	“femgay”	Living with HIV, started and stopped ART more than once.	Informal hairstylist	Moves in between formal and informal houses of extended family, usually sharing a room or staying in an informal extended room.	R3400 (US$227) per month	Internal family (mother and brother) moved away, therefore she moves between houses of extended family members (niece or aunt) or friends.
Sizwe	21	Black	Xhosa	“MSM”	HIV negative & no other physical health concerns	Informal work in retail	Has her own room in a shared three bedroom brick house.	R2500 (US$167) per month	Lives with her mother (**consented**), sister and brother.
Steven	28	Coloured	Afrikaans	“gay” or “femgay”	Living with HIV. Starts and stops ART regularly. Previous TB diagnosis. Went for drug rehabilitation.	Formal retail job	Has her room in a shared informal wooden house in aunt’s backyard.	R7350 (US$490) per month	Lives with her sister, mother (**consented**) and father (**consented**). Her aunt (consented) lives in the main house.
Owen	25	Coloured	Afrikaans	“transgender woman”	Both her and her sister are living with HIV and both are on consistent ART.	Unemployed, rents out house	Own bedroom in a shared three bedroom brick house.	R1500 (US$100) per month	Lives with her sister in a house where a lot of friends of them come to have casual sex during the day. Sometimes they rent the house out and stay with friends.
Patricia	32	Coloured	Afrikaans	“gay”	HIV negative & casual drug user.	Sex work	Moves between informal houses. Shared an informal garage space previously. Has been homeless in the past.	R3000 (US$200) per month	Stays currently on an open piece of land in a self-erected informal house of her own, with her friend (**consented**) and friend’s husband (**consented**) as neighbours.
Girlie	27	Coloured	Afrikaans	unable/ unwilling to categorise	Living with HIV. Casual drug user which is why clinic staff doesn’t want her to start ART.	Sex work & street hawking	Shares a room with her brother in a shared two bedroom brick house. Sometimes sleeps outside the house when boyfriend visits.	Information not available	Stays with brother (**consented**) and mother. Her step-sister (consented, but stopped participation) and extended family are next door neighbours.

Our team consisted of graduate social and behavioural science researchers, from different disciplinary backgrounds like psychology, sociology, anthropology and political studies. The researchers were trained on ethnographic interviewing skills in the beginning of 2016 by the last author (LR), who is a trained and experienced ethnographer. A graduate researcher was responsible for the data collection with a participant and their household unit. The researcher was always accompanied in the field by a research assistant, for safety reasons. The first three authors (LdV, AT, DJ) were involved with recruitment and data collection with all eight participants. The longitudinal nature of the cohort built a relationship of trust among the researchers and the participants. This allowed for open conversation around sensitive topics and participants could inform the researchers at any stage when they felt uncomfortable and researchers could provide participants with additional support information and contact details.

### Data analysis

We conducted a case-comparative narrative analysis on the data of the eight participants and their households. We chose this analytic approach because of the diversity and the large number of sources of data, including recordings, images, scanned activity sheets, videos, fieldnotes and reflection documents. The narrative approach helped us unpack the intricate stories of people as they developed over the time researchers spent with them and their household members. This was an inductive process of filtering through data to identify parts that revolved around the main objectives: stigma, gender identity and HIV services. Ayres, Kavanaugh & Knafl [28] explain that, “the generalizations developed by qualitative researchers are embedded in the contextual richness of individual experience” [28]. A narrative analysis approach allowed us to think with complete narrative accounts about the participants and to see these narratives as rooted in subjective experiences of participants in a specific time and space [29].

Analysis was conducted by LdV, AT, DJ and LR. We followed a similar approach to the narrative analysis approach of Riessman [29], whereby a separate narrative piece is constructed for each participant based on all available data. Data collection ended in August 2017, after which LdV reviewed all of the data of participants to prioritise transcriptions. This process was done because recordings were not all relevant to the life story of the participants, as some were family members talking about topics unrelated to the participant. Ninety recordings (approximately 82 h) were identified as relevant to the analysis presented here, which included interviews with family members as well. Eighty-three recordings were recorded and transcribed in Afrikaans, a language in which LdV is fluent. Seven recordings were in Xhosa and were transcribed and then translated into English.

LdV read through all the data (transcripts, fieldnotes, activities documents and reflection documents) of each participant and took detailed notes to develop the narrative or story of each participant. The initial analysis process involved within-case analysis, focused on the data of each person and their social networks. Based on this, LdV wrote detailed narrative descriptions of the ‘life stories’ of each of these eight people. These drafts were shared with co-authors who led on the data collection with these participants, allowing comments and edits to the narrative. This process was repeated for each of the eight participants’ datasets. These eight narratives were then examined comparatively to understand the diversity of participants’ experiences of stigma and accessing HIV services. The comparisons involved the analysis team having detailed discussions and noting important similarities and differences amongst the participants.

## Results

The first part of the findings describes participants’ unique gender experiences and how they anticipated, experienced and internalised gender identity-related and HIV-related stigma. We then present data on how transfeminine and gender diverse women experienced stigma related to different and intersecting parts of their identity. The final part of the findings describes how stigma affects HIV service access (including testing, treatment and adherence support and services) for the participants and potential ways in which participants were able to manage stigma.

### The transfeminine and gender diverse women in our study

Table 1 shows a breakdown of the participants’ pseudonyms, their age, ethnic group, language, identity category, HIV and health status, employment, as well as housing type, household income and household member structure and setup.

All of the participants reported being assigned male sex at birth and having feminine gender identifications, with predominantly feminine gender expressions. They used various feminine pronouns and gender expressions to refer to themselves. During data collection, only one of the eight participants identified as a transgender woman (Owen). The other participants had diverse ways of expressing their gender and sexual identities. Some of the participants, such as Stacey, Steven and Curella, for example, used the term “femgay” and feminine pronouns to identify themselves. Stacey explained: *“femgay is me, twenty-four-seven, female-looking, female-acting, female me”* (6 June 2016). Sizwe distinctly uses the term “MSM”.

Participants described fluid and diverse ways in which their gender identifications functioned in different social settings. They expressed their gender as feminine in most spheres of their lives, including in romantic and sexual relationships. However, they reported being addressed at times by their masculine, birth given names, mainly by family members and people from the community. Participants often used terms connected to their sexual identity (see Table 1).

All our participants are from urban study communities on the periphery of Cape Town city. Some of them live in detached houses on open pieces of land, others in shared farm labourer houses or in informal township neighbourhoods. All but one of the participants were Afrikaans-speaking coloured^2^ people.

Participants had diverse household structures. Steven, Curella, Girlie, Georgina and Sizwe live with their nuclear families that included at least one sibling and one parent. These households often included extended family members like aunts, uncles and cousins who lived inside the house, next door or close by. Stacey and Patricia had more transient household structures, as they would often move from one house to another and struggled with familial ties. Owen and her sister lost their parents and are now living together and sharing their house with friends and peers who socialise there often.

The participants shared income with their household members, with combined household earnings ranging from ZAR 1500 to ZAR 9000 (which is about 100 USD to 600 USD). According to Statistics South Africa [31], in 2015, 30,4 million South African, or about half of the total population were living under the national poverty line of just below ZAR 1000 (about USD 67) per person per month. Considering that participants stayed and shared income with household members of 5 to 10 people (and sometimes even more), all of the participants lived under the poverty line. All had financial constraints and had experienced times when they didn’t have enough food to eat for them and their households. Girlie and Patricia would revert to sex work, whereas other participants relied on family, friends and peers to share income and food in times of financial hardship.

Four participants (Stacey, Girlie, Owen and Steven) disclosed that they were living with HIV. Owen was the only one who was taking ART consistently. Stacey and Steven reported having initiated but stopped using ART regularly. Girlie had never started on ART because of her health worker’s concern about her drug use. Patricia was also a consistent drug user and Steven had been to a rehabilitation centre recently because of her drug addiction. Steven has experienced several TB disease episodes.

Participants did not necessarily use terms related to stigma. Instead, we identified and labelled forms of stigma implicit in the participants’ experiences and dialogue. Below we discuss instances of anticipated, experienced and internalised gender identity-related stigma.

### Gender identity-related stigma

Participants anticipated that revealing aspects of their gender identity or the way they live their lives might lead to negative consequences. For example, Girlie, was very concerned about exposing her gender identity to her family, who might find out she cross-dresses, and to her sex work clients, who might find out she is assigned male sex at birth if they approached her on the premise of being a cisgender woman.

Some participants also anticipated negative reactions around specific sexual partners, identified as ‘after-niners’ – These are men who are known to some in the communities they live as cisgender men who present publicly as ‘straight’ during the day, but who secretly indulge in sexual encounters with feminine gay and transgender women at night [32]. For Sizwe, ‘after-nine’ partners potentially put her at risk of being exposed to stigma and discrimination, and so she anticipated stigma related to her gender identity when interacting with these men:

*“Most of them are my friends, but also [have sex with] others, yes. But then maybe it is dependent on me and then if I am satisfied maybe with sex, then I don’t mind we continue. Even at the club if I see you, there’s no need to make it obvious. I do my own things and you do your own things but don’t make people look at us”.* (Sizwe, 8 November 2016)

In an earlier discussion she explained that people often recognise her as a woman:

*“When I come through they see a lady, so not unless uh not unless I show my ID or something that shows I’m a male, then they will be shocked”* (Sizwe, 8 November 2016).

Sizwe anticipated a negative reaction in terms of shock or surprise in someone’s demeanour. For other participants, this anticipated negative reaction to their gender identity might be fear of a verbal insult or physical attack, as Conry explained:

“But if you walk around here, then you’ll: “Aye look at THAT *moffie* and what and so and so”. (Conry, 29 August 2016).**”**

All participants could recall having had negative experiences of stigma and discrimination due to their gender identity and gender expression at some point during their lives. Girlie described experiencing stigma and discrimination from young men and gangsters who would emotionally and physically abuse her because she presented herself as a woman. For example, in one incident, she was assaulted by young boys in an open field. The boys hurled insults and threats at her, like *“Look, there goes that moffie*^3^”, and “*Let’s go and rob him”*, and then they chased her and beat her up. The other sex workers (who are cisgender women) also got angry when clients were interested in Girlie, and she told us they would sometimes instigate assaults on her.

“Y*es, then those girls maybe say: ‘I don’t like that thing [Girlie]. That thing takes all our ‘ways’ [potential partners] away from us and so and so’ ... then those [women] will probably stay down here and work other people up to hurt me”* (Girlie, 4 August 2016).

Participants also described experiencing verbal abuse linked to their gender identity, including being called derogatory names and insults such as “*moffie*”, “*moffie fuck*”, “*that thing*”, *“fucked through the ass*” and “*stabane*”.^4^ There was evidence that stigmatising terms participants were called affected them negatively. Steven, in particular, explained how her father would want to buy her a toy car instead of the doll she wanted when she was young. She seemed to connect some negative attitude to it, as she explained that she knew that she “*wasn’t right*” or normal.

### HIV stigma

In addition to gender identity-related stigma, participants’ narratives also included examples of HIV stigma. All four participants who disclosed they were living with HIV explained that they had anticipated and experienced stigma related to their HIV status. Many of the experiences of HIV-related stigma were interconnected with their gender or sexual identity. In a discussion about people who she has told about her HIV status, Girlie anticipated everyone to believe that she was living with HIV, although she had not told anyone except for one close friend:

“*No I didn’t tell everyone, but you know how people are, right? They like to tell it over [gossip]*” (Girlie, 31 August 2017).

Stacey said that her HIV status is a “state secret” that she only shared with a close friend. She reiterated the secrecy of her HIV status throughout the visits. Negotiating her undisclosed HIV status was always a challenge for the research team, as it was unclear who might know or who might not know her status. This meant that the research team had to keep her secret of living with HIV from others. Below we show an example of how Stacey anticipated stigma related to her HIV status (14 September 2017):

Researcher: *Why don’t you yet want to tell people? [about your HIV status]*

Stacey: *[Breathes in deeply] Well, I think if you tell people then they l-look at you differently they treat you differently. You get your own plate, you get your own mug, your own fork, your own spoon, your own everything.*

Three out of the four participants living with HIV (Steven, Girlie and Stacey) had disclosed only to one or two close friends or close family members and not to anyone else. Almost all the participants had experienced people in the community speaking in a negative and stigmatising manner about people living with HIV, as reflected in the following conversation with Georgina’s aunt:

“*Because in the community it is just like this. If you are HIV positive, then you are bad*” (Georgina’s aunt, 10 August 2017).

Curella also explained the different ways that a person living with HIV might be labelled and stigmatised by community members:

“*There are these nicknames for HIV, it will be Aunty Vera*^5^*or groot griep [“great*

*flu*^6^*”]. Those are the names that are used*” (Curella, 11 August 2017)*.*

### Multiple barriers to care

Our narrative analysis highlighted that our participants have intricate lives in which multi-layered social identities further complicate how they anticipate, experience and internalise stigma. HIV stigma and gender identity stigma often overlap, as Stacey explained:

“*People think in general, uh, that people like us, my sort, are those people who spread the virus. And it’s not what I feel, but what I know; I have heard it. Not for me, obviously, but talking about someone else*” (Stacey, 26 October 2016).

Apart from their gender identity, all of the participants were also faced with stigma and discrimination related to other parts of their social identities like same-sex sexual orientation and sex work. Girlie’s brother explained how, despite caring about her well-being, he doesn’t agree with her way of life. He also referred to her as a man and not a woman:

“*It is almost like this, the way how he is, almost like this. I am not really turned against it, but the things he does isn’t right for me ... this that he now has ‘n “berg” [boyfriend] and what what. It isn’t right, I am turned against it*” (Girlie’s brother, 24 January 2017).

Our interactions with participants over the duration of the cohort helped us understand the complexities of their stigmatised lives. Steven and Stacey had to navigate stigma related to their feminine gender identity as well as potential stigma related to living with HIV. For Girlie and Patricia, intersectional stigma could be very complicated. For example, they both are assigned male sex at birth but identify as women, identify and are stigmatised as homosexual men, do highly stigmatising work (sex work), have been incarcerated, use drugs and live very transient lifestyles. For Girlie, this intersects further with stigma she experiences related to her positive HIV status.

### How stigma affects HIV service access for participants

All participants, especially the ones who disclosed to be living with HIV, described staff at the local health facility that were accepting and open towards their gender identity. Despite this, health facilities were highly stressful and potentially stigmatising spaces for participants, as people could see that they accessed specific HIV services, like counselling rooms or clinic pharmacy windows. Stacey, for example, was really distraught about accessing ART services at her local clinic:

“*Because the silly/ridiculous fact that you have to walk to the clinic and have to go in at a specific room, is already a hold up for me. To stand caught up because then I must first look who else is at the clinic and who will now see in and that.* (Stacey, 26 October 2016)

Owen avoided testing at the clinic initially, even though she felt sick at times,

“*I was too scared of what they were going to say, what they going think of me and how my reactions would be when I get out of the clinic because most of the people will see on your face that ‘oh he’s HIV positive or oh no he’s not’, you see*” (Owen, 5 September 2017).

Stacey and Steven have both been living with HIV for a couple of years and they often struggle with taking ART consistently. Both of these women experienced barriers to HIV services in terms of treatment uptake and sustained treatment because they anticipated experiencing stigma if their HIV status became known. Stacey and Steven struggled to stay on their medication, as none of their household members were aware that they are living with HIV. They therefore tried to secretly take their medications and hide their pills from household members, as Steven explained:

“*My pills are in their original container but I once wanted to throw it in a bag so that it doesn’t make such a noise when I drink my pills in the evenings*” (Steven, 14 September, 2017).

When Stacey was on medication, she used to hide her medication under her bed. She described that:

“*Usually I pull off the sticker [label] with my name on, because if you throw the container away, everyone knows what this bottle looks like, right, and what pills go in it, so if they see my name on it then my secret is now out*” (Stacey, 26 October 2016).

The personal and social lives of participants made it hard for them to stay on their treatment. For example, when Steven was out socialising and drinking, she would not take her medication, and similarly, when she was sleeping over or if she was away for the weekend with friends she would choose to not take her pills with her.

### How participants coped with stigma

Participants mentioned becoming accustomed to experiencing stigma related to their gender identity. Below, Curella explained what it was like growing up identifying as different than her assigned sex at birth:

*“Eh it was rather a bit heavy. Not heavy but [pauses] if I’m with children, especially when they make remarks. Then I did feel rather bad, because [pauses] they would call it [make remarks] like that non-stop. That’s why today I now, I don’t worry about it anymore, because I know this now already [have become used to the remarks]. It has been coming on for years now, but that time I did [worry] a bit, because I was in my shell, very quiet still [pauses] withdrawn* (Curella, 29 August 2016).

Despite the participants becoming accustomed to negative reactions from people in their communities, we found that social support often counteracted negative reactions towards participants’ gender identity. This included social support and acceptance from a broader community, internal social networks like family and friends, support organisations and social support from peers and friends within their close communities. When it came to acceptance from the broader community, participants explained that, with time, community members were able to better accept them. In a way, people became used to and accustomed to the participants living in the community, as Georgina and Curella explained:

“*As I became more involved in the community, people started to love me for what I am*” (Georgina, 17 May 2017),

“*They [the community] understand, And I realised that they are now used to me.*” (Curella, 11 August 2017).

Apart from the community members in general becoming more accustomed to the participants’ gender identity, social support within the participants’ inner social networks also seemed constructive to their social well-being. Below, Georgina’s family member explained:

“*It’s just plain and simple. He feels he wants to be that person. The lord created him ... the lord created us, right? He didn’t ask to ... he was like that from a young age. He didn’t ask to be a moffie. Like he has female hormones*” (Georgina’s family member, 17 August 2016).

Sizwe explained that her mother supported her the most when she was still a child:

“*Because she saw me from the start that I liked girl things. So she had to buy those things and when I said “mom, I want this” she would buy it .. whether a dress or ...*” (Sizwe, 8 Novemebr 2016).

Steven also experienced positive support from her family:

“*My parents were now very ... they had very open thoughts and they are very open-minded. They accepted me just as I am ... actually it was never necessary for me to tell them. They knew*” (23 August 2016).

Other social support was also provided by organisations within the community regarding stigma related to gender and sexual identity. Two participants, Patricia and Simone, described receiving support from local non-profit organisations that offer emotional support and treatment services for the Lesbian, Gay, Bisexual, Transgender, Queer and Intersex (LGBTQI) community:

“*I joined where everyone, where the lesbians and the gays and the bisexuals and the transgender people can meet each other and where we have a safe space here in this area/community*” (Simone, 29 September 2016).

Participants also described the supportive nature of close relationships with peers and friends who also have fluid gender identifications. Curella, Stacey, Steven and Simone are all part of a small sub-culture of feminine-bodied men who take part in cross-dressing modelling shows. This community is supportive of one another and enables people to cope with shared experienced stigma related to gender identity. Curella explained that these modelling shows instilled courage in her to walk in front of others and show off her ‘true self’. She also explained that these shows created a sense of community amongst peers and friends who clearly are not always accepted for who they are:

“*I think it’s a good thing tha’ more people can know [pauses] that, how can I say, that more people understand what it’s about. Because a lot of people don’t get used to the idea that gay people exist or so*” (Curella, 11 August 2017).

## Discussion

Through the lived descriptions of the transfeminine and gender diverse women in the study, we identified additional barriers to care that intersect with HIV stigma including low socio-economic status/class (being unemployed or having low income); marginalised jobs like sex work; negatively viewed social behaviours like drug use and having committed a crime (sometimes because of sex work); as well as a negative view towards institutionalised rehabilitation of criminals or drug users (including incarceration and accessing a rehabilitation centre). Other international studies have also reported types of intersectional stigma (like stigma related to trans−/homophobia, stigma related to gender or sexism, racism and sex work) and described how HIV stigma exacerbates these stigmas [12, 36].

Public health researchers have started to try and understand the highly oppressed and stigmatized lives of transfeminine and gender diverse women through intersectionality. Bowleg [8] posits intersectionality as a theoretical framework where multiple micro level social categories (gender, sex, race, ethnicity, socio-economic status) intersect with multiple interconnected macro level social-structural oppression (racism, sexism, heterosexism). It is apparent that transfeminine and gender diverse women face stigma and discrimination because of multiple overlapping social identities. We recommend that future research with similar groups of women use intersectionality as a framework to better develop interventions that are sensitive to gender identity, sexual orientation and other social identities that may affect HIV stigma.

There is complexity in the way that gender diverse persons in our study expressed their gender identity, that varied from person to person and was different from their sexual orientation (feminine men who have sex with other men). Part of this problem is connected to the way gender and sexuality of gender diverse and transgender people are misconstrued or conflated. A recent special issue of Global Public Health aimed to provide a more balanced understanding of sexual and gender diversities and re-evaluate approaches to HIV prevention and health promotion [37]. The editors argued that research in public health should move beyond a focus on epidemiological risk practices and behaviours and instead explore the unique lived experiences of gender and sexual minorities in local contexts [37].

This study is foregrounded by important work from the likes of Donham [38], which problematized already in the 1990s how traditional South African cultural beliefs connected same-sex attraction (sexual identity) and cross-dressing (gender identity or expression) to intersex behaviour. With more recent studies in South Africa with black men who have sex with men [39], we found that gender identity and sexual orientation are not mutually exclusive, but rather intimately connected. For a large part of the recent history of Africa, homosexuality, as well as transgender identity, has been construed as a Western concept brought by colonialisation [40]. Conflation of gender and sexuality is common in the African context, where religious and political conservatism often limit expression of gender to a binary level [39, 40].

Transfeminine and gender diverse women in our sample all expressed anticipated and experienced stigma related to their gender identity. The use of words like *stabane* and *moffie* were used by community and family members of the participants to described effeminate men in the study. Derogatory labels are often connected to social stereotypes and discriminatory categories for people [41]. A person who is labeled or called by a derogatory name may not internalise the label, but often may incorporate parts of the label into their identity [42]. Similar to our results, a South African study of transgender female and cisgender male sex workers found that social identities can contribute to social exclusion [43]. Hegemonic masculine identities then often ostracise transfemininities and gender diverse identities as “failed masculinities” and “illegitimate femininities” [43].

Future research should investigate the unique gender identifications of transgender women, transfemine and other feminine gender diverse persons and how they relate to HIV service access, specifically in a South African context. In order for local research on this highly stigmatised and under-researched group to develop into more quantitative based studies, we need to first understand how ‘transgender’ as a collective term is understood in the local context. How do transgender and gender diverse women describe their gender identity and sexual orientation? What unmet needs does this group have? How are people who identify as women, who are assigned male sex at birth and who don’t intend on transitioning, excluded from transgender-focused HIV services and general population-based services? We suggest that more work should initially be done on understanding some of these difficult issues and for interventions to be community-led.

It was evident from our results that participants used social support networks as a way of coping with stigma, whether related to HIV, gender identity or other intersectional stigma. Other studies have also found resilience factors like community and personal relationships used by transgender people to counteract stigma [10, 44]. We therefore recommend that future research engage more on fostering resilience in the form of social support networks, and delve into understanding how transfeminine and gender diverse women are able to renegotiate new systems of support when family support structures fail them. Future research should also investigate intersectional stigma further among transfeminine and gender diverse populations by examining other potential intersections with race, sex work, drug use, incarceration and class.

The data for this study are from a small sample that was largely homogenous. However, the open-ended and longitudinal nature of the interviews resulted in rich encounters of gender fluidity amongst a group of people who are experiencing intersectional stigmas that affect their risk for HIV and how they engage with HIV services. Our research highlights the need for more nuanced work (potentially longitudinal in nature) amongst a highly stigmatised group of people. Our sample presented a glimpse into the transfeminine and gender diverse experience in a South African context, but the country has many different racial, cultural and religious groups, which future studies should include.

We recommend that researchers on future studies with similar groups of women be highly sensitive to the individuals and families that participate in the study. Because of the types of stigma expressed by participants in our study, especially the way they are easily identified in their communities if they are known to be transfeminine and gender diverse, participants might avoid being seen to participate in HIV-related research. All participants, except for one, were open for us to involve their families and households in the research. The one participant, Girlie, preferred us to do interviews with her at designated spots away from her home. This is difficult and resource intensive work, but we believe it is much needed amongst a group of women who are often misunderstood and often discriminated against.

South Africa provides a unique challenge for offering health services to transfeminine and gender diverse women because of poor resources, which make highly specialised care like gender-affirming services, along with specialised HIV services, problematic. For the participants in our study, as well as other transfeminine and gender diverse women, the main and probably only avenue for seeking health services is at general and centralised heath facilities. We therefore recommend that HIV programmes must engage the diverse and shifting ways people understand and negotiate their gender identities and the way they access HIV services. This might mean gender diversity training programmes that help health service staff understand diverse gender identifications and sexual orientations [45]. Instead of designing programmes for specific risk groups, the aim should rather be on a more inclusive training package or interventions that sensitise staff to gender fluidity.

## Conclusion

Our study found that transfeminine and gender diverse women living in communities in the Western Cape of South Africa anticipated, experienced, and internalised stigma due to their gender identity, HIV status and other intersecting stigmas linked to drug use, sex work and MSM identities. Stigma interfered with HIV testing, engagement with the healthcare system, and adherence to antiretroviral treatment. While experienced stigma reinforced perceived life limitations, such as not being able to have open relationships with partners, several sources of emotional support that existed within families and communities were reported.

## Supplementary Information

**Additional file 1.** Topic guide_20201113. The file includes topic guide themes used for the interviews in the larger qualitive cohort study.

## Data Availability

The data used for this manuscript form part of a databank for the HPTN071 (PopART) study, which can be made available by application and request to the trial working group.

## Abbreviations

ART: Antiretroviral therapy
HIV: Human Immunodeficiency virus
ID: Identification document
LGBTQI: Lesbian, Gay, Bisexual, Transgender, Queer and Intersex
MSM: Men who have sex with men
STI’s: Sexual transmitted diseases
TB: Tuberculosis
USD: United States Dollar
ZAR: South African Rand

## Footnotes

1 Failure to document the existence of someone’s gender identity [1]

2 Coloured was originally a racial classification used during South Africa’s Apartheid period, but it is used today to describe a racial and cultural category, typically referring to people of mixed raced descent. We use the term sensitively in a South African context and people would rather be asked whether they identify with the term or not, than just assuming their racial category [30]..

3 Derogatory Afrikaans word for a gay or effiminate man. Mainly used as a slang word with possible roots from the Dutch “mofrodiet” for ‘hermaphrodite’ [33, 34]

4 A derogatory Zulu word, meaning ‘two organ’ or ‘hermaphrodite’, often used for gay and lesbian persons [35]

5 *Aunt Vera* is a term used among gay and effeminate women in Curella’s community, where the V for Vera refer to ‘virus’.

6 *Great flu* is a more generally used Afrikaans term to speak of HIV or someone who is living with HIV.

## References

[1] Bauer GR, Hammond R, Travers R, Kaay M, Hohenadel KM, Boyce M (2009). “I don’t think this is theoretical; this is our lives”: how erasure impacts health care for transgender people. J Assoc Nurses AIDS Care [Internet].

[2] Fletcher JB, Kisler KA, Reback CJ (2014). Housing status and HIV risk behaviors among transgender women in Los Angeles.

[3] Kaplan RL, Wagner GJ, Nehme S, Aunon F, Mokhbat J, Kaplan RL (2015). Forms of safety and their impact on health : an exploration of HIV / AIDS-related risk and resilience among trans women in Lebanon. Health Care Women Int.

[4] Baral SD, Poteat T, Strömdahl S, Wirtz AL, Guadamuz TE, Beyrer C (2013). Worldwide burden of HIV in transgender women: A systematic review and meta-analysis. Lancet Infect Dis.

[5] Bao A, Colby DJ, Trang T, Le BQ, Dinh TD, Nguyen QH (2016). Correlates of HIV testing among transgender women in Ho Chi Minh, Vietnam. AIDS Behav.

[6] White Hughto JM, Reisner SL, Pachankis JE. Transgender stigma and health: A critical review of stigma determinants, mechanisms, and interventions, vol. 147: Social Science and Medicine; 2015. 10.1016/j.socscimed.2015.11.010 PMC4689648 26599625

[7] Andrinopoulos K, Hembling J, Guardado ME, de Maria Hernández F, Nieto AI, Melendez G. Evidence of the negative effect of sexual minority stigma on HIV testing among MSM and transgender women in San Salvador, El Salvador. AIDS Behav [Internet]. 2015 Jan 8 [cited 2018 May 22];19(1):60–71. Available from: http://link.springer.com/10.1007/s10461-014-0813-0. 10.1007/s10461-014-0813-0 24907779

[8] Arístegui I, Radusky PD, Zalazar V, Lucas M, Sued O. Resources to cope with stigma related to HIV status, gender identity, and sexual orientation in gay men and transgender women. J Health Psychol [Internet]. 2018 Feb 26 [cited 2018 may 18];23(2):320–31. Available from: http://www.ncbi.nlm.nih.gov/pubmed/29069922. 10.1177/1359105317736782 29069922

[9] Woodford MR, Chakrapani V, Newman PA, Shunmugam M (2016). Barriers and facilitators to voluntary HIV testing uptake among communities at high risk of HIV exposure in Chennai, India. Glob Public Health [Internet].

[10] Perez-Brumer AG, Reisner SL, McLean SA, Silva-Santisteban A, Huerta L, Mayer KH (2017). Leveraging social capital: multilevel stigma, associated HIV vulnerabilities, and social resilience strategies among transgender women in Lima. Peru J Int AIDS Soc.

[11] Logie CH, Wang Y, Lacombe-Duncan A, Jones N, Ahmed U, Levermore K, et al. Factors associated with sex work involvement among transgender women in Jamaica: a cross-sectional study. J Int AIDS Soc [Internet]. 2017 [cited 2018 May 18];20. Available from: https://www-ncbi-nlm-nih-gov.ez.sun.ac.za/pmc/articles/PMC5515035/pdf/zias-20-1309862.pdf. 10.7448/IAS.20.01/21422 PMC5515035 28406598

[12] Logie CH, Ll J, Tharao W, Loutfy MR (2011). HIV, gender, race, sexual orientation, and sex work: A qualitative study of intersectional stigma experienced by HIV-positive women in Ontario, Canada. PLoS Med.

[13] Evans MGB, Cloete A, Zungu N, Simbayi LC (2016). HIV risk among men who have sex with men, women who have sex with women, lesbian, gay, bisexual and transgender populations in South Africa: a mini-review. Open AIDS J.

[14] UNAIDS. UNAIDS: Country, South Africa, Overview [Internet]. 2018 [cited 2018 May 25]. Available from: http://www.unaids.org/en/regionscountries/countries/southafrica.

[15] SANAC. Let our actions count: South Africa’s National Strategic Plan for HIV, TB and STIs 2017–2022 [Internet]. 2017 [cited 2019 Feb 1]. Available from: http://sanac.org.za//wp-content/uploads/2017/06/NSP_FullDocument_FINAL.pdf.

[16] Müller A (2016). Health for all? Sexual orientation, gender identity, and the implementation of the right to access to health care in South Africa. Health Hum Rights [Internet].

[17] Müller A. Scrambling for access: Availability, accessibility, acceptability and quality of healthcare for lesbian, gay, bisexual and transgender people in South Africa. BMC Int Health Hum Rights [Internet]. 2017 [cited 2018 May 18];17. Available from: https://www-ncbi-nlm-nih-gov.ez.sun.ac.za/pmc/articles/PMC5450393/pdf/12914_2017_Article_124.pdf. 10.1186/s12914-017-0124-4 PMC5450393 28558693

[18] Wilson D, Marais A, De Villiers A, Addinall R, Campbell M. Transgender issues in South Africa, with particular reference to the Groote Schuur Hospital Transgender Unit. South African Med J [Internet]. 2014 [cited 2018 May 31];104(6). Available from: http://journals.co.za.ez.sun.ac.za/docserver/fulltext/m_samj/104/6/m_samj_v104_n6_a30.pdf?expires=1527759907&id=id&accname=57845&checksum=B476FFD88E217D0D842BF30069C2669E. 10.7196/samj.8392 26301294

[19] Nkoana T, Nduna M. Thematic focus: Transgender Issues - Engaging primary health care providers in transgender community health care, Observations from the field. Psychology [Internet]. 2012 [cited 2018 May 31];8(2):120–9. Available from: http://journals.co.za.ez.sun.ac.za/docserver/fulltext/unipsyc/8/2/unipsyc_v8_n2_a10.pdf?expires=1527755279&id=id&accname=57845&checksum=5FC3A6998B8BF794D4E6C0162F5D8F7C.

[20] Luvuno Z, Ncama B, Mchunu G. Knowledge, attitudes and practices of health care workers related to treatment and care of transgender patients: A qualitative study in Kwazulu-Natal, South Africa. Gend Behav [Internet]. 2017 [cited 2018 May 31];15(2):8694–706. Available from: http://journals.co.za.ez.sun.ac.za/docserver/fulltext/genbeh_v15_n2_a19.pdf?expires=1527760453&id=id&accname=57845&checksum=599152575741AB3FDF44FBF622393D1B.

[21] Smit DM, Viviers D. Gender reassignment and the world of work: A comparative perspective on the intersection between transgenderism, trans- sexuality and appearance discrimination in the South African employment arena. Obiter [Internet]. 2016 [cited 2018 May 31];37(2):247–64. Available from: http://journals.co.za.ez.sun.ac.za/docserver/fulltext/obiter/37/2/obiter_v37_n2_a3.pdf?expires=1527760538&id=id&accname=57845&checksum=89B43029FFDD8B48976D9EDEB57E907E.

[22] Matthyse G. Heteronormative higher education: Challenging this status quo through LGBTIQ awareness-raising. South African J High Educ [Internet]. 2017 [cited 2018 May 31];31(4):112–26. Available from: http://journals.co.za.ez.sun.ac.za/docserver/fulltext/high_v31_n4_a7.pdf?expires=1527760484&id=id&accname=57845&checksum=160D14D6C7E90AA7F547189F4E0D7B51.

[23] Gower AL, Rider GN, Brown C, McMorris BJ, Coleman E, Taliaferro LA, et al. Supporting Transgender and Gender Diverse Youth: Protection Against Emotional Distress and Substance Use. Am J Prev Med [Internet]. 2018 [cited 2019 Jun 25];55(6):787–94. Available from: https://pdf.sciencedirectassets.com/271902/1-s2.0-S0749379717X00172/1-s2.0-S0749379718321299/main.pdf?x-amz-security-token=AgoJb3JpZ2luX2VjEMn%2F%2F%2F%2F%2F%2F%2F%2F%2F%2FwEaCXVzLWVhc3QtMSJHMEUCIQDTluw%2B4m3G9q1blf82wgMdrjDlfSichGzxGzu1U%2B8LggIgQ9GtqoQn. 10.1016/j.amepre.2018.06.030 PMC6501838 30344037

[24] Hayes R, Ayles H, Beyers N, Sabapathy K, Floyd S, Shanaube K, et al. HPTN 071 (PopART): Rationale and design of a cluster-randomised trial of the population impact of an HIV combination prevention intervention including universal testing and treatment - a study protocol for a cluster randomised trial. Trials [Internet]. 2014 Feb 13 [cited 2020 Sep 5];15(1):57. Available from: http://trialsjournal.biomedcentral.com/articles/10.1186/1745-6215-15-57. 10.1186/1745-6215-15-57 PMC3929317 24524229

[25] Hargreaves JR, Stangl A, Bond V, Hoddinott G, Krishnaratne S, Mathema H (2016). HIV-related stigma and universal testing and treatment for HIV prevention and care: design of an implementation science evaluation nested in the HPTN 071 (PopART) cluster-randomized trial in Zambia and South Africa. Health Policy Plan.

[26] Molina Y. Ramirez-Valles. HIV/AIDS stigma: measurement and relationships to psycho-behavioral factors in Latino gay/bisexual men and transgender women. AIDS Care [Internet]. 2013;25(12):1559–9 Available from: http://www.tandfonline.com. 10.1080/09540121.2013.793268 PMC3800251 23668809

[27] Reisner SL, Deutsch MB, Bhasin S, Bockting W, Brown GR, Feldman J (2016). Advancing methods for US transgender health research. Curr Opin Endocrinol Diabetes Obes [Internet].

[28] Ayres L, Kavanaugh K, Knafl KA. Within-Case and Across-Case Approaches to Qualitative Data Analysis. Qual Health Res [Internet]. 2003 Jul 1 [cited 2018 May 22];13(6):871–83. Available from: http://journals.sagepub.com/doi/10.1177/1049732303013006008. 10.1177/1049732303013006008 12891720

[29] Riessman CK, Van Maanen J, Manning PK, Miller ML (1993). Narrative analysis. Qualitative research methods series.

[30] Laubscher LR. Suicide in a South African town: A cultural psychological investigation. South African J Psychol [Internet]. 2003 [cited 2018 Oct 4];33(3):133–43. Available from: http://citeseerx.ist.psu.edu/viewdoc/download?doi=10.1.1.835.57&rep=rep1&type=pdf.

[31] Statistics South Africa. Poverty trends in South Africa: An examination of absolute poverty between 2006 and 2015 / Statistics South Africa [Internet]. 2017. Available from: www.statssa.gov.za.

[32] Mantell JE, Tocco JU, Osmand T, Sandfort T, Lane T (2016). Switching on after nine: black gay-identified men’s perceptions of sexual identities and partnerships in south African towns. Glob Public Health [Internet]..

[33] Luyt K. Gay language in Cape Town: A study of Gayle – attitudes, history and usage. 2014 [cited 2018 May 4]; Available from: https://open.uct.ac.za/bitstream/item/6868/thesis_hum_2014_luyt_k.pdf.

[34] Gevisser M, Cameron E. Defiant desire [Internet]. Ravan Press; 1994 [cited 2018 Nov 6]. 376 p. Available from: https://sun-primo.hosted.exlibrisgroup.com/primo-explore/search?query=any,contains,defiant desire&tab=default_tab&search_scope=default_scope&vid=27US_V1&lang=en_US&offset=0.

[35] Lock Swarr A. “Stabane,” intersexuality, and same-sex relationships in South Africa [Internet]. 2009 [cited 2018 Nov 6]. Available from: http://content.ebscohost.com/ContentServer.asp? T=P&P=AN&K=47191494&S=R&D=vth&EbscoContent=dGJyMNLr40SeprQ4zOX0OLCmr1CeprNSsK64TLCWxWXS&ContentCustomer=dGJyMPGqsEq0rrVPuePfgeyx44Dt6fIA.

[36] Dougherty DS, Rick JM, Moore P. Unemployment and social class stigmas. J Appl Commun Res [Internet]. 2017 [cited 2018 Jul 11];45(5):495–516. Available from: http://www.tandfonline.com/action/journalInformation?journalCode=rjac20.

[37] Parker R, Aggleton P, Perez-Brumer AG (2016). The trouble with ‘categories’: rethinking men who have sex with men, transgender and their equivalents in HIV prevention and health promotion. Glob Public Health [Internet].

[38] Donham DL (1998). Freeing South Africa: The “Modernization” of Male-Male Sexuality in Soweto. Vol. 13, Anthropology.

[39] Sandfort TGM, Bos H, Reddy V. Gender expression and mental health in black South African men who have sex with men: further explorations of unexpected findings. Arch Sex Behav [Internet]. 2018 Feb;47(8):2481–90. Available from: 10.1007/s10508-018-1168-9. 10.1007/s10508-018-1168-9 PMC6102085 29464453

[40] Theron LB, Kgositau TR (2015). The emergence of a grassroots African trans archives. Transgender Stud Q.

[41] Mupenda B, Duvall S, Maman S, Pettifor A, Holub C, Taylor E, et al. Terms used for people living with HIV in the Democratic Republic of the Congo. Qual Health Res [Internet]. 2014 [cited 2018 Oct 31];24(2):209–16. Available from: https://www.ncbi.nlm.nih.gov/pmc/articles/PMC4326230/pdf/nihms661098.pdf. 10.1177/1049732313519869 PMC4326230 24463633

[42] Victor JS. Sluts and wiggers: A study of the effects of derogatory labeling. Deviant Behav [Internet]. 2004 [cited 2018 Oct 30];25(1):67–85. Available from: http://www.tandfonline.com/action/journalInformation?journalCode=udbh20.

[43] Samudzi Z, Mannell J. Cisgender male and transgender female sex workers in South Africa: Gender variant identities and narratives of exclusion. Cult Health Sex [Internet]. 2016 Jan 2 [cited 2018 may 18];18(1):1–14. Available from: http://www.ncbi.nlm.nih.gov/pubmed/26242843. 10.1080/13691058.2015.1062558 26242843

[44] Bariola E, Lyons A, Leonard W, Pitts M, Badcock P, Couch M (2015). Demographic and psychosocial factors associated with psychological distress and resilience among transgender individuals. Am J Public Health.

[45] Poteat T, Park C, Solares D, Williams JK, Wolf RC, Metheny N, et al. Changing hearts and minds: Results from a multi-country gender and sexual diversity training. Newman PA, editor. PLoS One [Internet]. 2017 Sep 19 [cited 2020 Sep 6];12(9):e0184484. Available from: https://dx.plos.org/10.1371/journal.pone.0184484. 10.1371/journal.pone.0184484 PMC5604941 28926568

