# Supplementary material for: Stigma and HIV service access among transfeminine and gender diverse women in South Africa – a narrative analysis of longitudinal qualitative data from the HPTN 071 (PopART) trial
Source: BMC Public Health. 2020 Dec 10;20:1898. doi: 10.1186/s12889-020-09942-5 (PMC7727216; doi:10.1186/s12889-020-09942-5)
Supplement: Supplementary file 1 — Additional file 1. Topic guide_20201113. The file includes topic guide themes used for the interviews in the larger qualitive cohort study. [file 12889_2020_9942_MOESM1_ESM.doc]

HPTN 071 (POPART) QUALITATIVE COHORT TOPIC GUIDES

| **Module 1 – Place and space** |
| --- |

***Activity/Topic area 1*** *– Drawing a picture of ‘the community’ places*

***Activity/Topic area 2*** *– Annotating a floor-plan of their home and surroundings*

***Activity/Topic area 3*** *– A timeline of in and out this study community*

***Activity/Topic area 5*** *– A guided transect walk to see the sights*

***Activity/Topic area 6*** *– Discussing the HPTN 071 geographic representations of ‘the community’*

**.**

| **Module 2 – How they get by** |
| --- |

***Activity/Topic area 1*** *– Catch up, December spending and kinship map update*

***Activity/Topic area 2 –*** *Pie chart for family income*

***Activity/Topic area 3****– Pie-charts for family expenses*

***Module 3 – Love, sex and romance***

***Topic area 1-*** *Personal discussions about sexual relationships (one on one)*

***Topic area 2 –*** Understanding sex

***Activity 3 -*** Questionnaire

***Module 4 – Understandings of HIV and trial intervention experience***

***Topic area 1:*** *Checklist.*

***Topic area 2*** *– Popular understandings of HIV in the community*

***Activity/Topic area 3*** *– Familiarisation with and discussion of the 90-90-90 HIV care continuum*

***Activity/Topic area 4*** *– HIV testing and acceptability of home-based HIV services*

***Activity/Topic area 5****: Exploring community members’ understanding and experiences of linking to**ART and remaining in care*

***Module 5 - hopes, dreams, and aspirations***

***Topic Area 1*** *– Experiences of participating in the research cohort*

***Topic Area 2*** *– Sharing thoughts and inferences*

***Topic area 3*** *– Hopes, dreams, and aspirations (individual interviews)*

***Topic area 4*** *– Time to ... say goodbye (group discussion and end off)*
